# Rapid diagnosis of extra pulmonary tuberculosis by automated Xpert MTB/RIF assay

**DOI:** 10.1186/1471-2334-12-S1-P32

**Published:** 2012-05-04

**Authors:** Shirly Suzana, Baby Shalini, Priscilla Rupali, K Venkatesh, DJ Christopher, Joy S Michael

**Affiliations:** 1 Department of Microbiology, Christian Medical College, Vellore, India

## Introduction

Although extra pulmonary tuberculosis accounts for only 10-15% of tuberculosis in India, they have high morbidity and mortality because of lack of good diagnostic methods. One of the recent molecular methods namely the Xpert MTB/RIF is a fully automated real time PCR system that detects *M. tuberculosis* (Mtb) complex and rifampicin resistance directly from clinical samples. This assay has been extensively evaluated in the diagnosis of pulmonary tuberculosis. The purpose of this study was to test the efficiency of this assay for the rapid and reliable diagnosis of extra pulmonary tuberculosis (EPTB).

## Materials and methods

This was a pilot prospective blinded study done on 60 samples received in the department of Microbiology from patients with signs and symptoms of EPTB. All the samples were tested by Xpert MTB/RIF as well as routine conventional smear and culture.

## Results

Overall the sensitivity, specificity, PPV and NPV of Xpert MTB/Rif assay in detecting *M. tuberculosis* from clinical samples was 86%, 100%, 100% and 83% respectively.

In the drug susceptibility testing (DST) there was 100% correlation between rifampicin susceptibility pattern between Xpert MTB/RIF assay and DST by MGIT/LJ method. The mean turnaround time by Xpert MTB/Rif assay was 2 hours when compared to culture and DST by LJ and MGIT systems were 46 days and 15 days respectively.

## Conclusion

The Xpert MTB/RIF is a rapid and reliable diagnostic assay for detection of *Mycobacterium tuberculosis* and rifampicin resistance in EPTB samples.

